# An Aγ-globin G->A gene polymorphism associated with β^0^39 thalassemia globin gene and high fetal hemoglobin production

**DOI:** 10.1186/s12881-017-0450-3

**Published:** 2017-08-29

**Authors:** Giulia Breveglieri, Nicoletta Bianchi, Lucia Carmela Cosenza, Maria Rita Gamberini, Francesco Chiavilli, Cristina Zuccato, Giulia Montagner, Monica Borgatti, Ilaria Lampronti, Alessia Finotti, Roberto Gambari

**Affiliations:** 1 Department of Life Sciences and Biotechnology, Ferrara University, Via Fossato di Mortara 74, 44121 Ferrara, Italy; 2 Department of Medical Sciences - Pediatry, Ferrara University, Ferrara, Italy; 3 Department of Transfusional Medicine - ULSS 18, Rovigo, Italy; 4 Biotechnology Center, Ferrara University, Ferrara, Italy

**Keywords:** β-thalassemia, Fetal hemoglobin, LYAR, Aγ-globin gene polymorphism

## Abstract

**Background:**

Increase of the expression of γ-globin gene and high production of fetal hemoglobin (HbF) in β-thalassemia patients is widely accepted as associated with a milder or even asymptomatic disease. The search for HbF-associated polymorphisms (such as the XmnI, BCL11A and MYB polymorphisms) has recently gained great attention, in order to stratify β-thalassemia patients with respect to expectancy of the first transfusion, need for annual intake of blood, response to HbF inducers (the most studied of which is hydroxyurea).

**Methods:**

Aγ-globin gene sequencing was performed on genomic DNA isolated from a total of 75 β-thalassemia patients, including 31 β^0^39/β^0^39, 33 β^0^39/β^+^IVSI-110, 9 β^+^IVSI-110/β^+^IVSI-110, one β^0^IVSI-1/β^+^IVSI-6 and one β^0^39/β^+^IVSI-6.

**Results:**

The results show that the rs368698783 polymorphism is present in β-thalassemia patients in the 5’UTR sequence (+25) of the Aγ-globin gene, known to affect the LYAR (human homologue of mouse Ly-1 antibody reactive clone) binding site 5′-GGTTAT-3′. This Aγ(+25 G->A) polymorphism is associated with the Gγ-globin-XmnI polymorphism and both are linked with the β^0^39-globin gene, but not with the β^+^IVSI-110-globin gene. In agreement with the expectation that this mutation alters the LYAR binding activity, we found that the Aγ(+25 G->A) and Gγ-globin-XmnI polymorphisms are associated with high HbF in erythroid precursor cells isolated from β^0^39/β^0^39 thalassemia patients.

**Conclusions:**

As a potential explanation of our findings, we hypothesize that in β-thalassemia the Gγ-globin-XmnI/Aγ-globin-(G->A) genotype is frequently under genetic linkage with β^0^-thalassemia mutations, but not with the β^+^-thalassemia mutation here studied (i.e. β^+^IVSI-110) and that this genetic combination has been selected within the population of β^0^-thalassemia patients, due to functional association with high HbF. Here we describe the characterization of the rs368698783 (+25 G->A) polymorphism of the Aγ-globin gene associated in β^0^39 thalassemia patients with high HbF in erythroid precursor cells.

## Background

The β-thalassemias are relevant hereditary hematological diseases caused by nearly 300 mutations of the β-globin gene, leading to low or absent production of adult β-globin and excess of α-globin content in erythroid cells, causing ineffective erythropoiesis and low or absent production of adult hemoglobin (HbA) [1–5]. Increase of the expression of γ-globin genes and high production of fetal hemoglobin (HbF) in β-thalassemia patients is widely accepted as associated with a milder or even asymptomatic disease [6–8]. In several cases, high HbF expressing β-thalassemia patients do not need transfusion regimen and, consequently, chelation therapy [6–8]. This well recognized finding has prompted researchers to develop efficient HbF inducers for treating β-thalassemia patients expressing low levels of HbF [9–14]. On the other hand, the search for HbF-associated polymorphisms (such as the XmnI, BCL11A and MYB polymorphisms) [15–19] has recently gained great attention, in order to stratify β-thalassemia patients with respect to expectancy of the first transfusion, need for annual intake of blood, response to HbF inducers (the most studied of which is hydroxyurea) [20–22].

In consideration of the fact that several HbF-related polymorphisms probably act in synergy, the interest in finding novel HbF-related genetic biomarkers has remained high. This field of investigation, in addition to a clear interest in diagnostics and prognostics, might bring novel therapeutic options, in the case the polymorphism(s) is (are) associated with novel therapeutic markers. This field of research has identified several direct or indirect transcriptional repressors of γ-globin gene expression such as BCL11A, KLF1, MYB, Oct-1 [16–19].

In a recent paper Ju et al. [23] identified a putative novel nuclear protein repressor of γ-globin gene transcription, LYAR (human homologue of mouse Ly-1 antibody reactive clone). The LYAR DNA-binding motif (GGTTAT) was identified by performing CASTing (cyclic amplification and selection of targets) experiments [23]. Results of EMSA (electrophoretic mobility shift assay) and ChIP (chromatin immunoprecipitation) assays confirmed that LYAR binds a DNA region corresponding to the 5′-untranslated region of the Aγ-globin gene. Ju et al. formally demonstrated that LYAR is a strong repressor of human fetal globin gene expression in both K562 cells and primary human adult erythroid progenitor cells. Interestingly, LYAR was found to directly interact also with the methyltransferase PRMT5 which triggers the histone H4 Arg3 symmetric dimethylation (H4R3me2s) mark. Altogether, these data indicate that LYAR acts as a novel transcription factor that binds the γ-globin gene, and is essential for silencing the γ-globin gene [23].

The objective of this study was to investigate the presence of genetic variants in β-thalassemia patients potentially affecting the LYAR binding site and the possible association with the most common HbF-associated polymorphism, the XmnI polymorphism [18, 24, 25]. To this aim we focused our attention on β-thalassemic patients from the north-west Mediterranean area, in particular those carrying the β^0^39 and β^+^IVSI-110 thalassemia mutations, allowing to compare β^0^- and β^+^-genotypes. The genomic DNA from these patients was studied by full sequencing of both the Gγ- and Aγ-globin genes.

## Methods

### Patients

A total of 75 β-thalassemia patients were recruited for this study, including 31 β^0^39/β^0^39, 33 β^0^39/β^+^IVSI-110, 9 β^+^IVSI-110/β^+^IVSI-110, one β^0^IVSI-1/β^+^IVSI-6 and one β^0^39/β^+^IVSI-6 patient. The β-thalassemia patients have been recruited at Ferrara Hospital and Rovigo Hospital. The Declaration of Helsinki was followed for the collection of blood samples from β-thalassemia patients; furthermore, specific approvals by the Ethical Committees of Ferrara Hospital and Rovigo Hospital were obtained. All the β-thalassemia patients duly signed the informed consent form before blood sampling.

### Genomic DNA extraction, polymerase chain reaction (PCR) and DNA sequencing

The genomic DNA from β-thalassemia patients was extracted from 500 μL of whole blood using the QIAamp® DNA Blood Mini Kit (Qiagen, Hilden, Germany) as described in Bianchi et al. [15]. PCR amplification of β-, Aγ- or Gγ-globin genes and DNA sequencing methods used in this study have been previously described by Bianchi et al. [15]. The nucleotide sequences of the PCR primers are reported in Table 1. BMR Genomics (Padua, Italy) performed gene sequencing.

**Table 1:** PCR primers for amplification of β-, Aγ- and Gγ-globin gene sequences

Gene	Forward(F)/Reverse(R)	Nucleotide sequence
β-globin	βF1	5′-GTG CCA GAA GAG CCA AGG ACA GG-3′
	βR1	5′-AGT TCT CAG GAT CCA CGT GCA-3′
	βF2	5′-GCC TGG CTC ACC TGG ACA-3′
	βR2	5′-GTT GCC CAG GAG CTG TGG-3′
	βF3	5′-ACA ATC CAG CTA CCA TTC TGC TTT-3′
	βR3	5′-CAC TGA CCT CCC ACA TTC CCT TTT-3′
Aγ-globin	AγF	5′-TTT CCT TAG AAA CCA CTG CTA ACT GAA A-3′
	AγR	5′-TTG TGA TAG TAG CCT TGT CCT CCT CT-3′
Gγ-globin	GγF	5′-TTC TTA TTT GGA AAC CAA TGC TTA CTA AAT-3′
	GγR	5′-TTG TGA TAG TAG CCT TGT CCT CCT CT-3′

### Erythroid progenitors (ErPCs) from β-thalassemia patients

The two-phase liquid culture procedure was employed as previously described [26, 27]. The erythroid differentiation status of ErPCs was verified analyzing transferrin receptor (TrfR) and glycophorin A (GYPA) expression by FACS (fluorescence-activated cell sorting) using the BD FACScan™ system (Becton, Dickinson & Company, Franklin Lakes, NJ, USA) and anti-human CD71 (TrfR) FITC-conjugated antibody (Miltenyi Biotec GmbH, Bergisch Gladbach, Germany) and anti-human CD235a (GYPA) antibody PE-conjugated (Miltenyi Biotec GmbH) as described elsewhere [28, 29]. Production of hemoglobins was assessed by high performance liquid chromatography (HPLC) as described elsewhere [11, 28].

### Statistical analysis

The results reported in this paper are usually presented as average ± SD. The one-way ANOVA (ANalyses Of VAriance between groups) software was used for compare statistical differences between groups. The paired t test of the GraphPad Prism Software was used to obtain the *p* values. Differences were considered statistically significant when *p* < 0.05 (*) and highly significant when *p* < 0.01 (**) [28, 29].

## Results

### Presence of the rs368698783 (G->A) Aγ-globin gene polymorphism in β-thalassemia patients

**Fig. 1:**
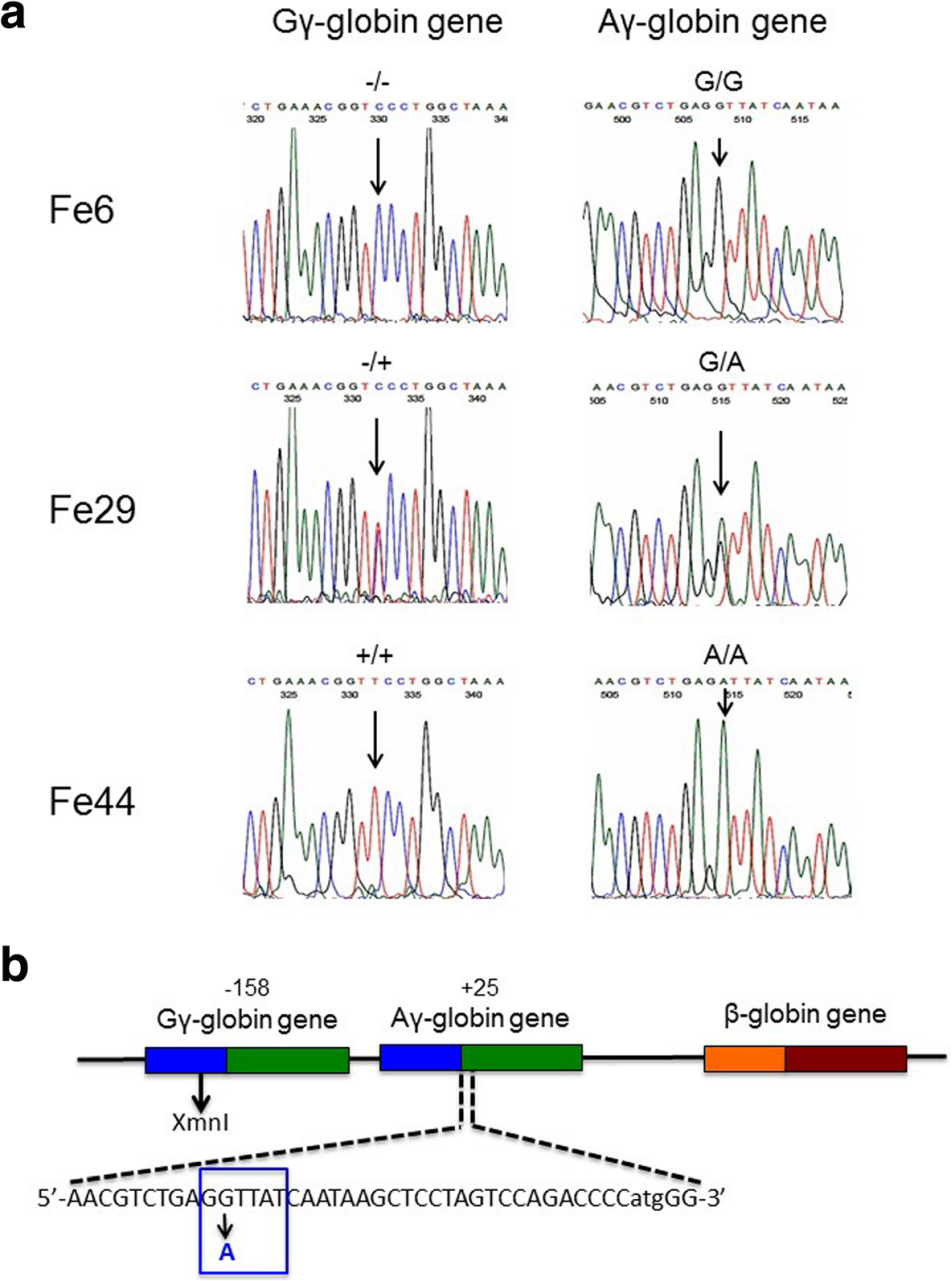
Sequence analysis of selected Aγ- and Gγ-globin genes. **a.** Right: representative sequence analysis of the Aγ-globin gene surrounding the region involved in LYAR binding site. The +25(G->A) polymorphism is arrowed. This corresponds to the already known rs368698783 polymorphism, which was not analyzed in full detail in the β-thalassemia patient population (including patients carrying β^+^ and β^0^ mutations). In the examples depicted the +25 Aγ-globin gene sequence is G/G (Fe6), G/A (Fe29) and A/A (Fe44). Left: the same genomic DNA has been sequenced at the XmnI site, found to be −/− in Fe6, −/+ in Fe29 and +/+ in Fe44 samples. **b.** Location of the −158 XmnI Gγ-globin and +25 Aγ-globin gene sequences within the Aγ- and Gγ-globin genes

In order to verify whether mutations affecting the LYAR-binding site of the Aγ-globin gene are present within our β-thalassemia patient population, sequencing of the Aγ-globin genes was performed using genomic DNA isolated from a total of 75 β-thalassemia patients, including 31 β^0^39/β^0^39, 33 β^0^39/β^+^IVSI-110, 9 β^+^IVSI-110/β^+^IVSI-110, one β^0^IVSI-1/β^+^IVSI-6 and one β^0^39/β^+^IVSI-6 patient. Examples of the sequencing results obtained are shown in Fig. 1a, which indicates that one (G->A) rs368698783 polymorphism was found in position +25 of the Aγ-globin gene, modifying the LYAR-binding sequence from 5′-GGTTAT-3′ to 5′-GATTAT-3′. For this reason, we called this mutation rs368698783 Aγ(+25 G->A) (see its location in Fig. 1b). In the examples reported in Fig. 1a, the representative homozygous (G/G), heterozygous (G/A) and homozygous mutated (A/A) genomic sequences are shown. In addition, as indicated in the representative examples shown in Fig. 1a, the G/G genotype is linked to the XmnI(−/−) haplotype; in contrast the G/A and A/A Aγ(+25) genotypes are linked to XmnI(−/+) and XmnI(+/+) haplotypes, respectively. Figure 1b shows the location of the mutation within the 5’UTR sequence of the Aγ-globin gene and the nucleotide change concerning the 5′-GGTTAT-3′ LYAR binding site proposed by Ju et al. Notably, no other nucleotide variations affecting the LYAR-binding sequence were found in these 75 patients. Moreover, no other mutations were found in the 607 bp and 613 bp sequenced regions of the Aγ-globin and Gγ-globin genes, respectively, with the exception of a 4 bp deletion residing in the promoter region of the Aγ-globin gene (HBG1: g.-225_-222delAGCA) [30], found in three XmnI(−/−), Aγ(+25 G/G) β^0^39/β^+^IVSI-110 patients. In 16/75 patients (21%) this Aγ(+25 G->A) polymorphism was found in the heterozygous (G/A) state, while the Aγ(+25) homozygous (A/A) state was found only in four patients. While we cannot exclude the presence of other mutations in the Aγ-globin genes of sub populations of β-thalassemia patients, we can conclude that the Aγ(+25 G->A) concerning the rs368698783 polymorphism is the most frequent mutation affecting this Aγ-globin gene region within our population, well representative of the Mediterranean area.

### The Aγ(+25 G->A) polymorphism is in complete linkage disequilibrium with the XmnI polymorphism

Table 2 shows that in all the patients analyzed the Aγ(+25 G->A) rs368698783 polymorphism is strictly linked to the Gγ-XmnI polymorphism. In fact all the 55 Gγ-XmnI(−/−) patients were found to be Aγ(+25 G/G). In addition, all the 16 Gγ-XmnI(−/+) patients were found to be Aγ(+25 G/A) and the four Gγ-XmnI(+/+) patients were found to be Aγ(+25 A/A). This very interesting distribution allows to hypothesize that the XmnI polymorphism, when present in this β-thalassemia patient population, is physically linked to the Aγ(+25 G->A) polymorphism.

**Table 2:** Distribution of the G->A Aγ-globin gene polymorphism and association with the XmnI Gγ-globin gene polymorphism in the β-thalassemia patients of this study

(G->A) mutation Aγ-globin gene			XmnI polymorphism Gγ-globin gene
G/G	A/G	A/A	
55	0	0	−/−
0	16	0	+/−
0	0	4	+/+

### Distribution of the Aγ(+25 G->A) polymorphism within the β^0^39/β^0^39, β^0^39/β^+^IVSI-110 and β^+^IVSI-110/β^+^IVSI-110 thalassemia patients

**Fig. 2:**
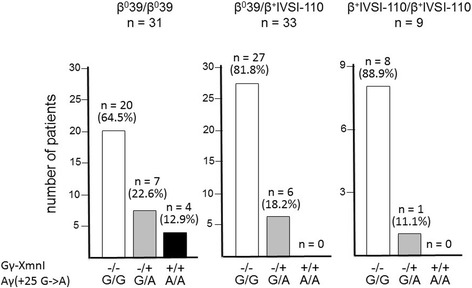
Distribution of the −158 XmnI Gγ-globin and +25 Aγ-globin gene polymorphisms within the studied β-thalassemia patients. The studied 73 patients were divided in β^0^39/β^0^39, β^0^39/β^+^IVSI-110 and β^+^IVSI-110/β^+^IVSI-110 subgroups and the −158 XmnI Gγ-globin and +25 Aγ-globin gene polymorphisms determined by direct sequencing

The results shown in Fig. 2 show that only one of the 9 β^+^IVSI-110/β^+^IVSI-110 patients was found to be Gγ-XmnI(−/+) and Aγ(+25 G/A) (11.1%). The other patients were Gγ-XmnI(−/−) and Aγ(+25 G/G) (88.9%). No patients exhibited a Gγ-XmnI(+/+) and Aγ(+25 A/A) combination. By sharp contrast, in the β^0^39/β^+^IVSI-110 cohort, the Gγ-XmnI(−/+) and Aγ(+25 G/A) patients were found to be 6 (18.2%), 27 being Gγ-XmnI(−/−) and Aγ(+25 G/G) (81.8%). Also in this case no patients exhibited a Gγ-XmnI(+/+) and Aγ(+25 A/A) combination. Finally, in the β^0^39/β^0^39 cohort the Gγ-XmnI(−/+) and Aγ(+25 G/A) patients were found to be 7 (22.6%), 20 being Gγ-XmnI(−/−) and Aγ(+25 G/G) (64.5%). Unlike the β^+^IVSI-110/β^+^IVSI-110 and the β^0^39/β^+^IVSI-110 cohorts, four β^0^39/β^0^39 patients exhibited a Gγ-XmnI(+/+) and Aγ(+25 A/A) combination (12.9%). These data, when analyzed together with the data shown in Table 2, support the hypothesis that the Gγ-XmnI and Aγ(+25 G->A) polymorphisms might be preferentially linked with the β^0^39 thalassemia mutation. To verify this hypothesis the family trees of all the available families with β-thalassemia patients carrying at least one β^0^39-globin gene were analyzed with respect to β-globin genes and Gγ-XmnI and Aγ(+25 G->A) polymorphisms.

### Association of the β^0^39 thalassemia mutation with Gγ-XmnI and Aγ(+25 G->A) polymorphisms

**Fig. 3:**
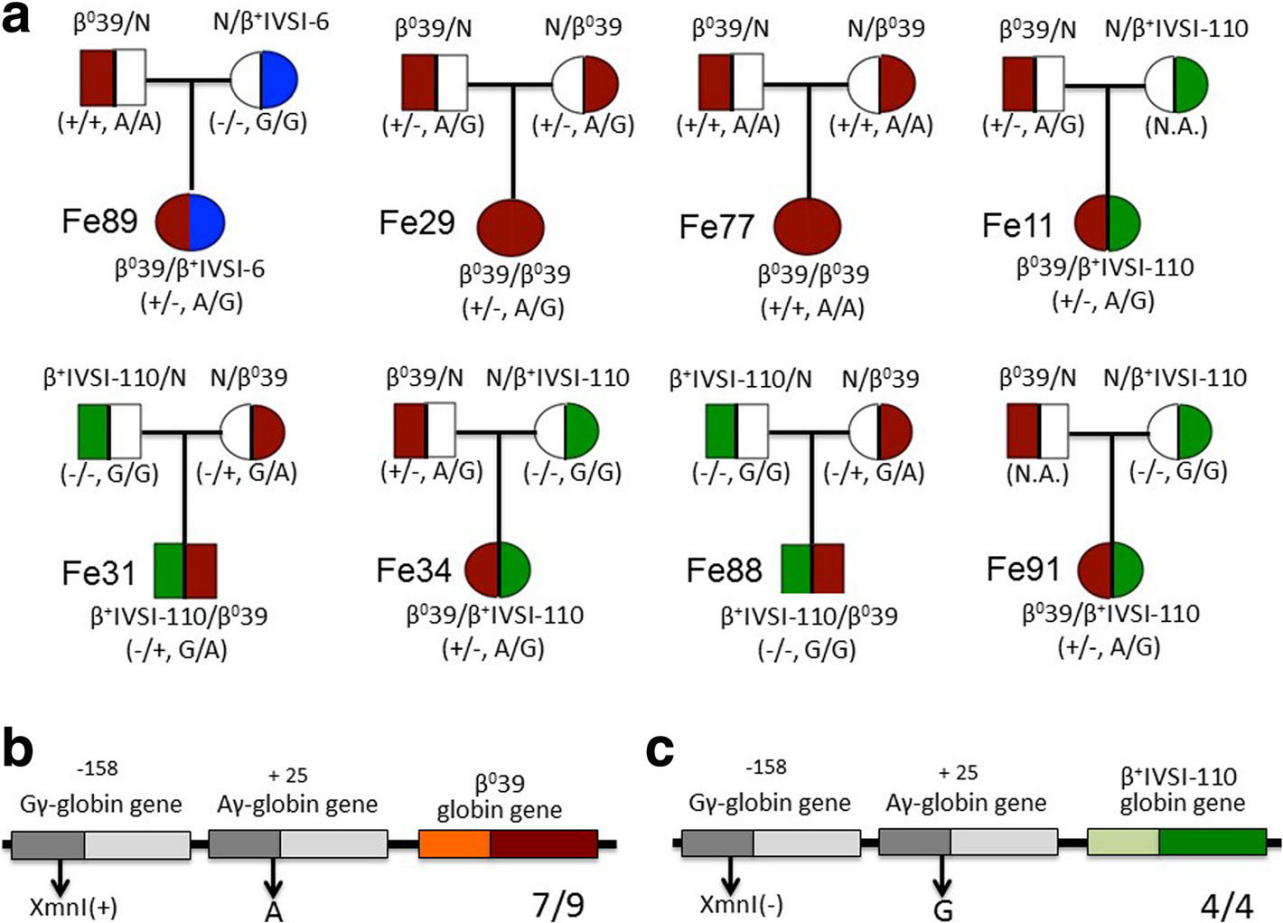
Genetic trees of the informative β-thalassemia families studied. **a** The analysis of the transmission of the −158 XmnI Gγ-globin and +25 Aγ-globin gene sequences from the parents to the studied β-thalassemia patients allowed to determine the linkage to the β^0^39 mutation. **b**, **c** Most frequent XmnI Gγ-globin and +25 Aγ-globin genes linked to β^0^39 (**b**) and β^+^IVSI-110 (**c**) globin genes. The comparative analysis does not include Fe11 because not informative. N.A. = not available

Figure 3a shows the family trees of a β^0^39/β^+^IVSI-6, two β^0^39/β^0^39 (out of three present in our cohort) and 5 β^0^39/β^+^IVSI-110 patients (out of the 6 available). The results obtained firmly demonstrate that in the β^0^39/β^+^IVSI-6 family (Fe89) the Gγ-XmnI and Aγ(+25 G->A) polymorphisms are linked to the β^0^39 gene (Fig. 3a, upper left side of the panel). In the two families with β^0^39/β^0^39 patients (Fig. 3a, upper middle side of the panel), one β^0^39-globin gene (Fe29) and both β^0^39-globin genes (Fe77) are associated with the Gγ-XmnI and Aγ(+25 G->A) polymorphisms. More importantly, when families with β^0^39/β^+^IVSI-110 were considered, one (Fe11) was not informative (the genome of the mother was not available), while in patients Fe31, Fe34, Fe88 and Fe91 the β^0^39 genotype was structurally linked to the Gγ-XmnI and Aγ(+25 G->A) polymorphisms combination. These polymorphisms were not associated with the β^+^IVSI-110 gene, as summarized in Fig. 3b. These data clearly indicate that in the population analyzed the Gγ-XmnI and Aγ(+25 G->A) polymorphisms cosegregate with the β^0^39-globin gene mutation in the majority of the families of compound-heterozygous β^0^-thalassemia patients.

### Production of fetal hemoglobin by erythroid precursor cells (ErPCs) from β^0^39/β^0^39 thalassemia patients: Relationship with Gγ-XmnI and Aγ(+25 G->A) polymorphisms

**Fig. 4:**
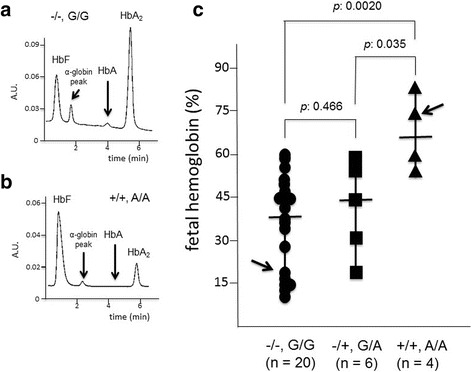
Relationship between the −158 XmnI Gγ-globin and +25 Aγ-globin gene polymorphisms and the level of fetal hemoglobin (HbF) in erythroid precursor cells from β^0^39/β^0^39 thalassemia patients. **a**, **b**. Representative HPLC analyses of the cytoplasms isolated from two ErPCs populations, one exhibiting low levels of HbF (**a**) and the other exhibiting high HbF levels (**b**) (arrowed in **panel c**). **c** HbF levels in ErPCs from 30 β^0^39/β^0^39 thalassemia patients (stratified on the basis of the 158 XmnI Gγ-globin and +25 Aγ-globin gene polymorphisms)

In order to verify possible relationships between the Gγ-XmnI and Aγ(+25 G->A) polymorphisms configuration, 30 available β^0^39/β^0^39 patients were recruited, peripheral blood isolated, erythroid precursor cells (ErPCs) selected and erythropoietin (EPO)-induced as elsewhere reported [27, 28]. After 7 days of EPO treatment, the erythroid differentiation was confirmed by benzidine-staining (looking at hemoglobin production) and FACS analysis of transferrin receptor and glycophorin A expression, as reported elsewhere [28] (data not shown). After demonstration that more than 80% of the cells were benzidine, TrfR and GYPA positive, HPLC analysis was performed to quantify fetal hemoglobin (HbF) production. Figure 4 (panels a and b) shows the representative HPLC profile of two ErPC lysates (arrowed in Fig. 4c), displaying a low (Fig. 4a) and high (Fig. 4b) HbF relative production. All the data obtained are shown in Fig. 4c, in which the ErPCs are stratified with respect to the Gγ-XmnI and Aγ(+25 G->A) polymorphisms configuration. As clearly evident, a significant correlation can be observed between the Gγ-XmnI and Aγ(+25 G->A) polymorphisms configuration and elevated production of HbF. In fact the average HbF production by ErPCs from β^0^39/β^0^39, Gγ-XmnI(+/+) and Aγ(+25 A/A) patients was 66.1 ± 14.1%, a value significantly higher than those found in ErPCs from Gγ-XmnI(−/−) and Aγ(+25 G/G) or Gγ-XmnI(−/+) and Aγ(+25 G/A) patients. This finding suggests that Gγ-XmnI and Aγ(+25 G->A) polymorphisms should be present in both alleles for maximal potentiation of HbF production, even if not “per se” sufficient and probably acting with other “HbF modifiers”, since all the Gγ-XmnI(+/+) and Aγ(+25 A/A) patients did not carry any detectable alteration of the α-globin gene asset. In any case, the cellular system here described might help to dissect genetic control of fetal-hemoglobin persistence and disease phenotypes, especially considering the possibility to access cellular biobanks from β-thalassemia patients stratified with respect to genotype, Gγ-XmnI and Aγ(+25) polymorphisms, enabling cryopreservation and usage of the cryopreserved and thawed cells for molecular biology studies [31].

## Discussion

Clinical observations have shown that increased levels of fetal hemoglobin (HbF) can ameliorate the severity of the disorders of β-hemoglobin, including β-thalassemia [7]. High HbF levels are associated with transcriptional effects on the γ-globin genes, which are associated with the biological activity of several transcription repressors, including MYB, BCL11A, Oct-1, KLF1 and others [16–19, 31–33]. A recent paper has pointed out the attention on a new putative repressor of the γ-globin gene, LYAR (human homologue of mouse Ly-1 antibody reactive clone), recognizing the Aγ-globin gene sequence 5′-GGTTAT-3′. Interestingly, several alterations within this consensus sequence for LYAR are associated with a decrease binding efficiency [23].

At present, no extensive analysis of this sequence has been reported in β-thalassemia patients; no attempts have been made to verify a possible association with the major HbF associated polymorphism, the Gγ-globin-XmnI; finally, no extensive analysis has been reported on possible linkage with β^0^- and β^+^-globin gene mutations.

In this paper we report the sequencing of the Aγ-globin genes performed on genomic DNA isolated from a total of 75 β-thalassemia patients, including 31 β^0^39/β^0^39, 33 β^0^39/β^+^IVSI-110, 9 β^+^IVSI-110/β^+^IVSI-110, one β^0^IVSI-1/β^+^IVSI-6 and one β^0^39/β^+^IVSI-6.

The major results of this paper are the following: (a) a G->A mutation at the level of the rs368698783 polymorphism is present in β-thalassemia patients in the 5’UTR sequence (+25) of the Aγ-globin gene, affecting the LYAR binding site 5′-GGTTAT-3′ sequence (Fig. 1); (b) no other mutations of the LYAR binding site were found; (c) this Aγ(+25 G->A) polymorphism is in complete linkage disequilibrium with a promoter variant of the Gγ-globin-gene (the XmnI polymorphism, rs7482144, C->T); (d) the Aγ(+25 G->A) and Gγ-globin-XmnI polymorphisms are linked with the β^0^39-globin gene, but not with the β^+^IVSI-110-globin gene (Figs. 2 and 3). Further genetic analysis in different β-thalassemia patient population is necessary (a) to extend this specific finding to other β^0^-thalassemia mutations and (b) to verify the link of Aγ(+25 G->A) and Gγ-globin-XmnI (C->T) polymorphisms with the β^0^39-globin gene in a statistically more significant number of patients.

## Conclusions

It is interesting to note that the Aγ(+25 G->A) rs368698783 polymorphism is expected to deeply alter the LYAR binding activity, thereby activating the Aγ-globin gene [23]. One possibility, which deserves to be verified in further studies, is that rs368698783, rather than the XmnI polymorphism, could be the physiologically (and even clinically) active variant in hemoglobinopathy patients carrying haplotypes including the XmnI(+) allele.

In respect to this point, our last conclusion is that the Aγ(+25 G->A) and Gγ-globin-XmnI polymorphisms might be associated with high HbF in erythroid precursor cells isolated from the β^0^39/β^0^39 thalassemia patients (Fig. 4), in agreement with several studies suggesting the association between XmnI polymorphism and high HbF production [18, 24, 34, 35].

On the other hand, as a potential explanation of our findings, we hypothesize that in β-thalassemia the Gγ-globin-XmnI/Aγ-globin-(G->A) genotype is frequently under genetic linkage with β^0^-thalassemia mutations, but not with the β^+^-thalassemia mutation here studied (i.e. β^+^IVSI-110). One hypothesis is the very interesting possibility that this genetic combination has been selected within the population of the β^0^-thalassemia patients, due to its functional association with high HbF.

## Abbreviations

BCL11A: B-cell lymphoma/leukemia 11A
EPO: erythropoietin
ErPCs: erythroid precursor cells
HbA: adult hemoglobin A
HbA2: adult hemoglobin A2
HbF: fetal hemoglobin
HPLC: high performance liquid chromatography
LYAR: human homologue of mouse Ly-1 antibody reactive clone
